# Radiochemoimmunotherapy with intensity-modulated concomitant boost: interim analysis of the REACH trial

**DOI:** 10.1186/1748-717X-7-57

**Published:** 2012-04-02

**Authors:** Alexandra D Jensen, Jürgen Krauss, Karin Potthoff, Christian Simon, Anna V Nikoghosyan, Karen Lossner, Jürgen Debus, Marc W Münter

**Affiliations:** 1 Dept of Radiation Oncology, University of Heidelberg, INF 400, 69120 Heidelberg, Germany; 2 Dept of Radiation Oncology, INF 400, 69120 Heidelberg, Germany; 3 National Centre for Tumour Disease (NCT), INF 460, 69120 Heidelberg, Germany; 4 Dept of Head and Neck Surgery, INF 400, 69120 Heidelberg, Germany

**Keywords:** Radiochemotherapy, Radioimmunotherapy, Cetuximab, IMRT, Concomitant boost, Squamous cell head and neck cancer, SCCHN

## Abstract

**Purpose:**

To evaluate efficacy and toxicity clinical in the intensified treatment of locally advanced squamous cell carcinoma of the head and neck (SCCHN) with the combination of chemotherapy, the EGFR antibody cetuximab, and intensity-modulated radiation therapy (IMRT) in a concomitant boost concept.

**Methods:**

REACH is a prospective, bi-centric phase II trial of carboplatin/5-FU and cetuximab weekly combined with IMRT. Primary endpoint is locoregional control, secondary endpoints include acute radiation effects and adverse events. Evaluation of disease response is carried out according to the Response Evaluation Criteria in Solid Tumors (RECIST); toxicity is assessed using NCI CTC v 3.0.

**Results:**

Treatment was tolerated moderately well, acneiforme erythema occurred in 74.1% (grade II/III), mucositis grade III in 28.6%, and radiation dermatitis grade III in 14.3%. Higher-grade side-effects resolved quickly until the first follow-up post treatment. Objective response rates were promising with 28.6% CR at first follow-up and 92.9% thereafter.

**Conclusion:**

The combination of standard carboplatin/5-FU and cetuximab is feasible and results in promising objective response rates. The use of an IMRT concomitant boost is practicable in a routine clinical setting resulting in only moderate overall toxicity of the regimen.

**Trial Registration Number:**

ISRCTN87356938.

## Introduction

Long-term disease control for patients with advanced squamous cell carcinoma of the head and neck (SCCHN) is still challenging. For locally advanced or unresectable SCCHN without evidence of distant metastases, combined radiochemotherapy is the only curative treatment possibility. In the MARCH meta-analysis, concurrent chemoradiotherapy results in an absolute survival benefit of 6.5% at 5 years with the most effective regimen being simultaneous chemoradiation [1-3]. This effect is mainly caused by an improved local control and only to a lesser extent by reduction of distant metastases. In the MARCH data, platin-based regimens were shown to have the highest effect in concurrent chemoradiotherapy [1-3].

Intensified radiotherapy such as altered fractionation schedules also lead to a significant improvement of local control but also overall survival if radiation therapy is performed as a single therapy modality with the highest benefit in hyperfractionated radiotherapy schedules [4]. Adding chemotherapy to altered fractionation radiotherapy also resulted in improved outcome [5].

While intensified treatment regimen - whether as intensified chemoradiation or altered fractionation radiotherapy - have been shown to improve outcome [4,6], it is a clinical fact that a significant percentage of patients are not able to receive their full planned course of treatment due to excessive toxicity, hence the need arises to modify and optimize these regimens.

Various options exist: first of all, the use of more tolerable chemotherapy combinations, second the integration of molecular targeted drugs and third the use of modern concepts of radiotherapy.

Staar et al. combined carboplatin with 5-FU and hyperfractionated accelerated radiotherapy in a randomized phase III trial [7] and presented comparable results to studies based on cisplatin. The published acute and late toxicity was moderate in this trial. Targeted therapy approaches with the EGFR antibody cetuximab have been found to potentiate the effects of chemotherapy and radiotherapy in experimental systems, Bonner and co-workers were the first to establish combined radioimmunotherapy for SCCHN in a definitive setting [8,9]. Overall survival and local control were significantly improved in the combination regimen as opposed to the radiotherapy-alone arm. Moreover, no further severe side effects were reported apart from acneiforme skin reactions and a low rate of infusion reactions [8,9]. Retrospective comparison of the Bonner data with landmark radiochemotherapy studies suggests comparable results could be achieved with this new combination [10].

Modern radiotherapy techniques such as intensity-modulated radiotherapy (IMRT) and image guidance (IGRT) have rapidly found acceptance in the routine treatment of SCCHN. Intensity-modulated radiotherapy (IMRT) has been shown to reduce acute and late toxicity [11] and prevent long-term sequelae such as higher-grade xerostomia by improved normal tissue sparing and preservation of saliva flow [12,13]. Improved normal tissue sparing permits relative dose escalation to the tumor in order to improve local control and patient outcome [14].

REACH combines all of these approaches: systemic treatment is intensified by the combination of chemotherapy and EGFR antibody treatment (carboplatin/5-FU and cetuximab) while local treatment is intensified by a concomitant boost concept in IMRT technique.

Aim of the trial is to evaluate disease control, toxicity, and feasibility of this intensified triple-therapy.

## Methods

### Patients

Patients with pathologically confirmed, locally advanced non-metastatic squamous cell cancer of oropharynx, hypopharynx, or larynx were eligible for the trial. Age between 18 and 70 years, Karnofsky performance score of > 70%, and adequate bone marrow, liver, and renal function were also required. Exclusion criteria were nasopharyngeal carcinoma, prior chemotherapy, radiotherapy, or exposure to EGFR pathway targeting therapy [15].

Work-up included complete panendoscopy, diagnostic CT scans of the neck and chest, abdominal ultrasound, and bone scan. In the absence of contraindications, all patients received diagnostic MRI scans for treatment planning and follow-up.

The trial was reviewed and approved by the University of Heidelberg Medical School Ethics Committee; informed consent was obtained from all patients prior to inclusion.

### Immunotherapy

Patients received the loading dose cetuximab one week prior to RT (d1) at the recommended dose of 400 mg/m^2 ^body surface followed by weekly infusions with 250 mg/m^2^. All patients received 8 mg dexamethasone and 4 mg dimetinden prior to each application of cetuximab. Missed out doses of cetuximab during the treatment course are to be omitted. Cetuximab-induced skin reactions are treated according to standard in-house protocols and recommendations of the vendor with topic or systemic antibiotics as indicated.

### Chemotherapy

Carboplatin and 5-FU are given on days 8-12 and 36-40 (corresponding to radiotherapy-week 1 and 5). Carboplatin is prescribed at 70 mg/m^2 ^body surface as a one-hour intravenous infusion, 5-FU at 600 mg/m^2 ^of body surface as continuous infusion over 23 hours. Patients are provided with standard antiemetic prophylaxis and hydration according to institutional protocols.

### Treatment planning and radiotherapy

Patients are immobilized using individual thermoplastic head masks incl. shoulder fixation (HeadStep^®^, ITV), planning examinations include CT-scan and contrast enhanced MRI for 3D image correlation. Target volumes are delineated in accordance with current guidelines and recommendations [16-18].

All patients are treated with inversely-planned intensity-modulated radiotherapy (IMRT) either at a 6 MV linear accelerator in step-and-shoot technique or at a 6 MV tomotherapy unit under regular image guidance.

A dose of 50.4 Gy in daily fractions of 1.8 Gy (Monday to Friday) is prescribed to the neck (CTV 2). From study day 29 onwards, patients receive an additional fraction at 1.5 Gy per day in a classical concomitant boost regimen [19,20] to a total dose of 69.9 Gy to the primary tumor and involved lymph nodes (CTV 1). There is at least a 6-h-interval between the two daily fractions.

### Follow-up

Regular follow up is carried out 6 weeks post treatment, 3 months (4-5 months post completion of therapy) thereafter, and then in 6 monthly intervals including fibreoptic examination and local imaging with MRI. In the presence of contra-indications, local imaging is carried out with contrast-enhanced CT scans.

### Study design and analysis

REACH is a prospective, bi-centric, single-arm phase II trial of combined radiochemoimmunotherapy with weekly cetuximab, carboplatin and 5-FU (according to the Staar protocol [7]) and IMRT in a concomitant boost concept. Planned accrual is 60 patients.

Primary endpoint of the trial is local-regional control (LRC); secondary endpoints are disease-free survival (DFS), progression-free survival (PFS), overall survival (OS), acute and late radiation effects, adverse events. Control and survival data are calculated from administration of cetuximab loading dose.

Physical examination and monitoring of adverse events as well as routine hematologic and chemical blood analysis is performed weekly throughout the treatment period. Toxicity is assessed using NCI CTC v 3.0.

Evaluation of disease response is carried out according to the Response Evaluation Criteria in Solid Tumors (RECIST) [21] 6 weeks, 4-5 months, 6-7 months post completion of treatment and then in 6-monthly intervals. Further details can be found in the published trial protocol [15].

## Results

Between August 2009 and March 2012, twenty-one patients were accrued to the REACH trial. Three patients subsequently had to be excluded from the trial: two patients had to be excluded due to poor compliance prior to treatment start, another patient developed an anaphylactic reaction to the trial medication on first exposure to cetuximab and had to discontinue treatment within the trial. Eighteen patients with a median age of 57 years (43 - 69) are treated within the protocol, fourteen patients have completed treatment. Most patients had very advanced disease not accessible to surgical treatment. Fifteen out of 18 patients were male, all patients had a history of heavy smoking. Fourteen patients completed trial treatment as scheduled. Median dose to the cervical lymph nodes was 50 Gy (49 - 51), the primary and involved lymph nodes received a median total dose of 70 Gy (69 - 74 Gy). Median follow-up from cetuximab loading dose excluding patients still under treatment (n = 14) is 17.4 months (6.5 - 30 months). Baseline characteristics are displayed in Table 1.

**Table 1:** Patient baseline and treatment characteristics; patients still under therapy are not included in follow-up/outcome analysis

characteristic		range
**median age (years), n = 18**	57	44 - 69

**median follow-up (months), n = 14 **excluding pts under treatment	17.4	6.5 - 30

deceased (pts)	1	

male (pts)	16	

female (pts)	5	

**Site (n = 18)**		

palate (pts)	2	

oropharynx	7	

hypopharynx	6	

larynx	3	

**Stage (n = 18)**		

T2	3	

T3	8	

T4	7	

N0	2	

N2b	4	

N2c	11	

N3	1	

**therapy (n = 14)**		

median dose (cervical lymph nodes)/Gy	50	49 - 51

median dose (primary and involved nodes)/Gy	20	19 - 24

median total dose/Gy	70	69 - 74

Treatment was generally tolerated well with the most common side effects being the typical, cetuximab-induced acneiforme skin rash, mucositis, dysphagia, dermatitis, and xerostomia. No chemotherapy cycle had to be postponed or dose-reduced due to toxicity, acneiforme rash was mostly moderate and did not lead to treatment interruptions or dose changes. Twelve out of 18 patients received a prophylactic feeding tube prior to therapy start, however, only one patient was feeding tube dependent during therapy. A list of observed side effects can be found in Table 2.

**Table 2:** Treatment adverse events (n = 14 pts)

adevers events (AEs), n = 14	severity (CTC v. 3) grade				
	**I**	**II**	**III**	**IV**	**V**

**acneiforme skin rash**	5 (35.7%)	9 (64.3%)	1 (7.1%)		

**conjunctivitis**		1 (7.1%)			

**skin fissures**	1 (7.1%)	1 (7.1%)			

**mucositis**	2 (14.3%)	8 (57.1%)	4 (28.6%)		

**dysphagia**	5 (35.7%)	7 (50%)	1 (7.1%)		

**weight loss**	1 (7.1%)	1 (7.1%)			

**dermatitis**	2 (14.3%)	6 (42.9%)	2 (14.3%)		

**xerostomia**	8 (57.1%)	5 (35.7%)			

**laryngeal oedema**	3 (21.4%)				

**anemia**		1 (7.1%)			

**leukopenia**			1 (7.1%)		

**thrombopenia**				1 (7.1%)	

**fever**	1 (7.1%)	1 (7.1%)			

**hyperkalaemia**	1 (7.1%)				

**hypokalaemia**			2 (14.3%)		

**hypomagnesiaemia**	3 (21.4%)				

**nausea**	5 (35.7%)				

**oedema**	7 (50%)				

**diarrhoea**	3 (21.4%)				

**constipation**	5 (35.7%)				

**Serious adverse events (SAEs)**					

**skin abscess**			1 (7.1%)		

**arterial embolism**			1 (7.1%)		

**septic shock**			1 (7.1%)		

**anaphylactic reaction**			1 (7.1%)		

**hospital admission due to tonsillitis**			1 (7.1%)		

**death due to progressive metastatic disease**					1 (7.1%)

There were six serious adverse events, three events were judged unrelated to the trial treatment: one patient has deceased due to systemically progressive disease (CTC °V), one patient was hospitalized for treatment of tonsillitis (CTC °III) 6 months after completion of therapy, and one patient developed septic shock of unknown genesis more than 6 months post completion of therapy, and had a protracted, complicated hospital stay. One patient was diagnosed with an abdominal wall abscess under therapy, which had also occurred several times in the past prior to trial treatment; relatedness to the trial medication is therefore questionable but cannot be excluded. Another patient developed an iliac artery embolism and consequently had to undergo surgery. This patient did not have any history of embolic events, no cardiac arrhythmias or any other predisposing factors. Relationship of this event with the trial treatment is therefore possible. There was one anaphylactic reaction to cetuximab at first exposure leading to discontinuation of trial treatment. All patients recovered from the adverse events.

Acute toxicity commonly observed in patients receiving chemoradiation is resolving rapidly so far, residual mucositis on the first follow-up (6 weeks post treatment) was seen in 7/14 patients. Dysphagia was generally resolving fast, 3 patients so far had evidence of dysphagia at the second follow-up. One patient with large mediastinal lymph node metastasis was still feeding-tube dependent though this is not attributable to study treatment. There is no higher-grade xerostomia, no evidence of higher-grade late effects have been found so far (Table 3).

**Table 3:** Resolution of treatment-related side effects on follow-up (n = 14)

typical side effects	CTC grade	end of treatment (N = 14)	6 weeks post completion of treatment, N = 14	4-5 months post completion of treatment, N = 14
**mucositis**	I	2 (14.3%)	5 (35.7%)	3 (21.4%)

	II	8 (57.1%%)	2 (14.3%)	

	III	4 (28.6%)	0	

**dermatitis**	I	2 (14.3%)	6 (42.9%)	

	II	6 (42.9%)	0	

	III	2 (14.3%)	0	

**acneiforme skin rash**	I	5 (35.7%)	1 (7.1%)	

	II	9 (64.3%)	0	

	III	1 (7.1%)	0	

**dysphagia**	I	5 (35.7%)	4 (28.6%)	

	II	7 (50%)	9 (64.3%)	3 (21.4%)

	III	1 (7.1%)	1 (7.1%)	1 (7.1%)

**xerostomia**	I	8 (57.1%)	9 (64.3%)	10 (71.4%)

	II	5 (35.7%)	2 (14.3%)	1 (7.1%)

**Figure 1:**
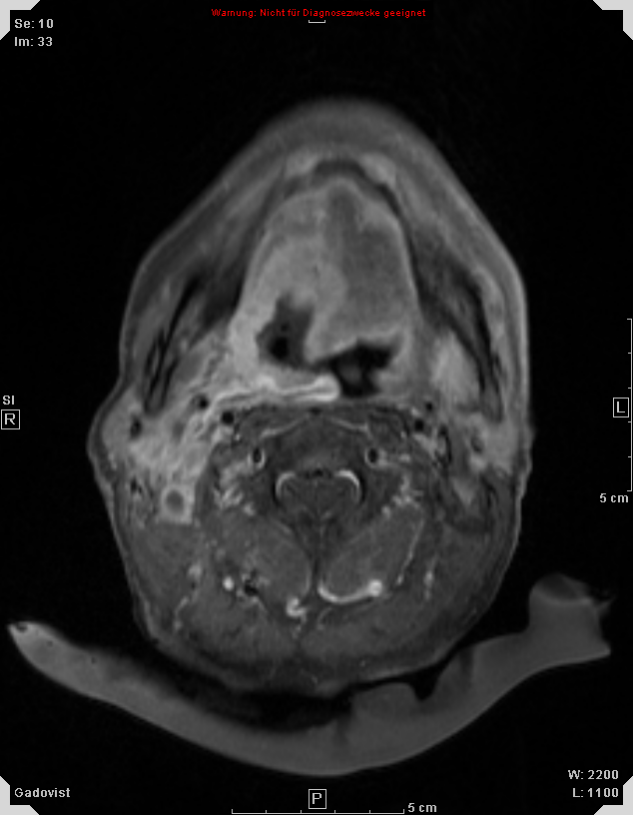
**50 y old patient with oro-/hypopharyngeal carcinoma; planning MRI**.

**Figure 2:**
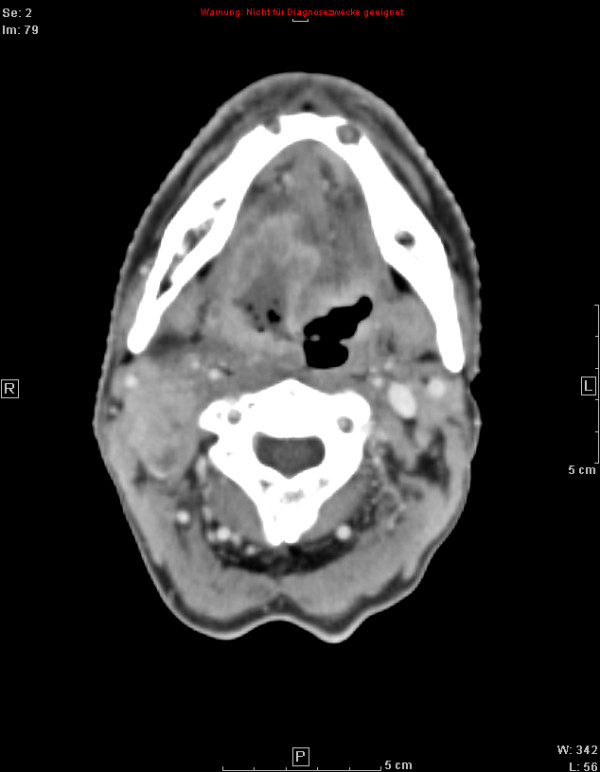
**50 y old patient with oro-/hypopharyngeal carcinoma; planning CT**.

**Figure 3:**
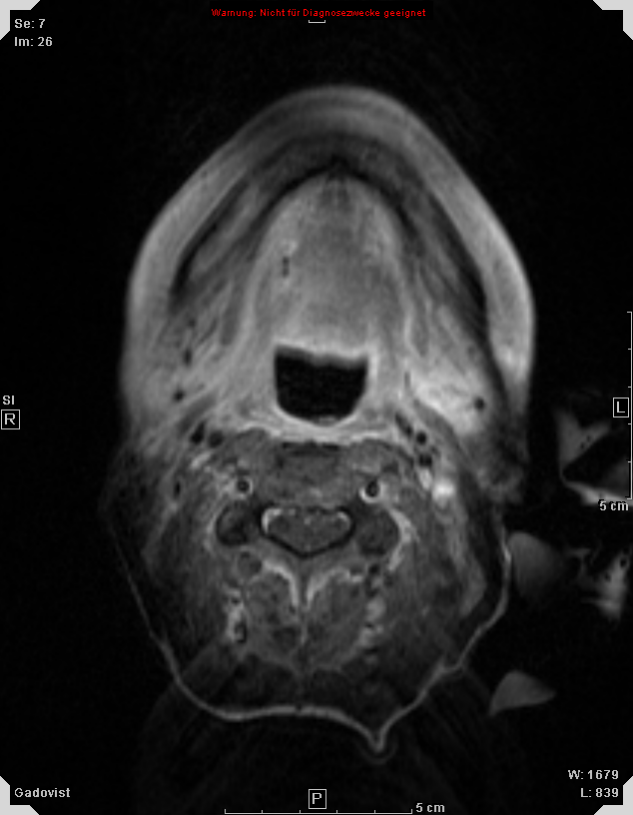
**50 y old patient with oro-/hypopharyngeal carcinoma; complete remission 6 weeks post completion of therapy**.

**Figure 4:**
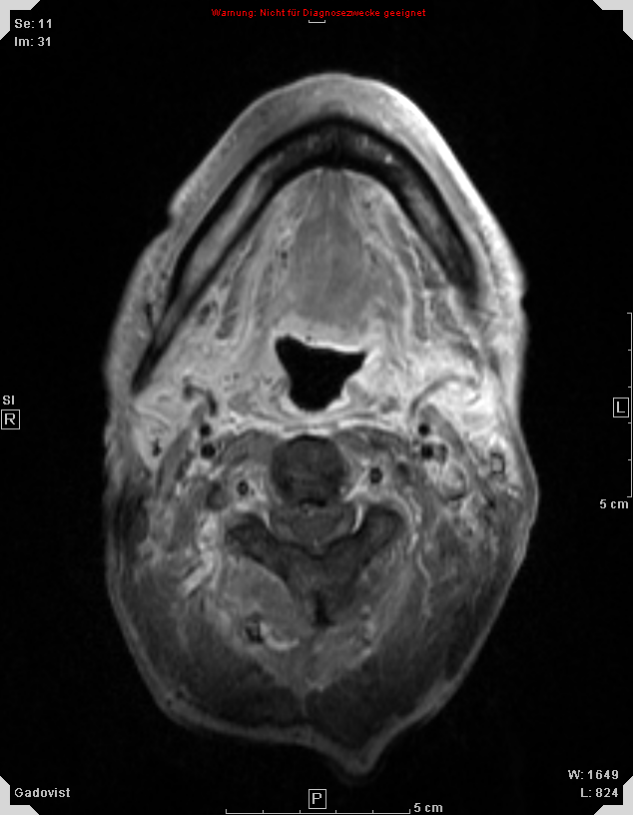
**50 y old patient with oro-/hypopharyngeal carcinoma; complete remission 4 months post completion of therapy**.

**Figure 5:**
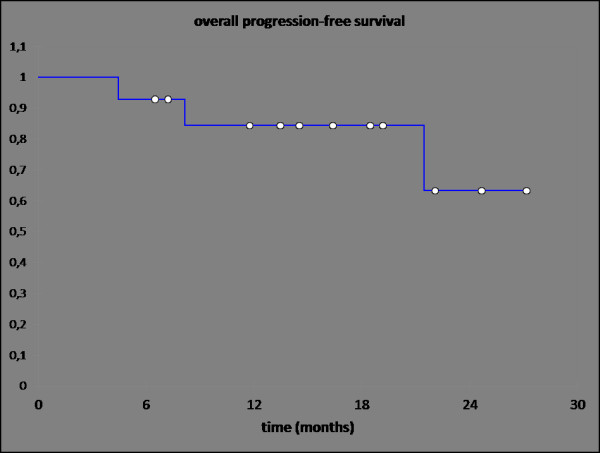
**Overall progression-free survival (PFS) (n = 14 pts)**. PFS at 1 year: 84.4%; PFS at 2 years 63.3%.

Treatment response is promising, first follow-up controls are available in 14 out of 18 patients treated within the protocol. Figures 1, 2, 3, 4 show a patient with a complete remission already at the first follow-up. While 4/14 patients showed complete remissions on their first follow-up, 8/14 patients had a CR on their second follow-up visit (approx. 4-5 months post completion of treatment). There was no planned prophylactic neck dissection after completion of treatment, one patient only has yet had to undergo neck dissection for residual nodal disease. Pathology of the neck dissection revealed no vital tumour cells. Six patients underwent microlaryngoscopy or panendoscopy and probe excision after a median of 5.5 months [2.9 - 26.3 months] post treatment. None of the samples showed any evidence of malignancy. Two patients only showed stable disease on their first follow-up. One developed a CR on his next visit, the other also had a distant disease progression and received palliative chemotherapy. This patient has deceased six months post completion of treatment. Only two further patients with a complete remission at the primary site developed distant disease (pulmonary metastases, osseous metastasis) after treatment, one received systemic chemotherapy according to the EXTREME regimen [22], the other received local irradiation for solitary osseous metastasis. Both are stable without further treatment (Table 4). Median progression-free survival (PFS) has therefore not yet been reached, overall progression-free survival at one year is currently 84.4% (Figure 5). Swallowing rehabilitation was successful in 13 out of the 14 patients, one patient had a persistent swallowing dysfunction due to distant metastatic disease with a large mediastinal mass.

**Table 4:** Treatment response

Local response	6 weeks post completion of treatment [pts, (%)], N = 14	4-5 months post completion of treatment [pts, (%)], N = 14	at time of analysis [pts, (%)] N = 14
**CR**	4 (28.6%)	8 (57.1%)	13 (92.9%)

**PR**	8 (57.1%)	5 (35.7%)	0

**SD**	2 (14.3%)	1 (7.1%)	1(7.1%)

**PD **(locoregional)	0	0	0

**PD **(distant metastases)	0	1 (7.1%)	3 (21.4%)

## Discussion

REACH combines the use of modern radiotherapeutic techniques (IMRT), altered fractionation (accelerated/hyperfractionated treatment), and systemic agents (concomitant radiochemotherapy with carboplatin/5-FU and the EGFR antibody cetuximab) in order to improve local control and ultimately patient outcome.

While treatment intensity with both the use of radiochemotherapy, immunotherapy, and concomitant boost is high, protocol treatment is tolerated reasonably well and without treatment delays. The combination of carboplatin and 5-FU has been shown to achieve similar results as cisplatin 100 mg/m^2 ^q3 weeks with a more favourable toxicity profile and subsequently higher patient compliance [7]. Bone marrow toxicity with grade III and IV (2/14 pts, 14.3%) was rare in our data and not higher than in our reference protocol [7]. Mucositis rates were moderate with no grade IV mucositis and 85.7% mucositis grade II and III. Observed mucositis rates are lower than in reported in the standard chemotherapy protocol where 68% mucositis grade III and IV were seen [7]. Mucositis rates in the landmark trials for definitive radiochemotherapy of locally advanced SCCHN vary widely between 12.8 and 77% grade III mucositis, most report rates roughly between 50 and 70% [7,23-27]. However, these trials mostly employed conventional radiotherapy techniques as IMRT was not the standard at the time, therefore our low mucositis rates may be attributable to the use of IMRT in our trial. Recent phase II trials also employing combined radiochemotherapy regimen and IMRT also report a lower incidence of grade III and grade IV mucositis between 29 and 38% [28-30]. Also, the rates of severe dysphagia of one in 14 patients (7.1%) and dysphagia CTC grade II (50%) in REACH is very low in comparison with rates published in the large phase III trials (31 - 44% dysphagia CTC grade III, [23,24,27]). Again, intensity-modulated radiotherapy trials report lower rates of severe dysphagia between 7% and 28% [28-30] supports the observation of the REACH trial.

Addition of cetuximab to chemoradiation with carboplatin/5-FU so far lead to a rather moderate, increased skin toxicity: acneiforme rash CTC grade II/III was present in 10/14 patients (71.4%) and correlates well with the observations by Bonner and colleagues [8,9] using conventional radiation techniques and 11 - 67% reported in the combined regimen using IMRT [29-31]. No cetuximab-specific exfoliations occurred so far. No grade IV and only one grade III cetuximab- induced skin toxicity was observed in the REACH protocol suggesting that our standard skin care protocols in accordance with the recommendations by the vendour help preventing major complications [32].

Response rates in the triple combination regimen are promising so far: two patients only had stable disease on their first follow-up after treatment corresponding to an overall objective response rate of 85.7% (CR: 28.6%, PR: 57.1%). One patient with stable disease had developed complete remission 4-5 months post completion of treatment, the other patient with stable disease on the first follow-up was diagnosed with distant metastases and has deceased 6 months post completion of the REACH regimen. REACH response rates are in the range of rates reported by other groups evaluating the combination of chemotherapy and cetuximab in the definitive setting [30,31,33] even though most of our patients had very advanced disease and therefore represent a very negative pre-selection. Interestingly though, theses protocols also carried out planned neck dissections irrespective of remission status post completion of treatment. Notably, most REACH patients showed partial remissions six weeks post completion of therapy and had developed complete remissions on the second follow-up three months afterwards reflecting the fact that tumour response may take longer than just 4-6 weeks after therapy. Our observation is supported by Beckmann and co-workers who saw only 16% complete remissions at the first follow-up and 73% thereafter [34]. Prevalence and influence of HPV in our cohort is currently under investigation.

We had six reported serious adverse events, for three of them there is (anaphylactic reaction) or may be a relationship to the trial medication (abdominal wall abscess, arterial embolism), all of these patients recovered uneventfully from the adverse event, hence discontinuation of the trial was not warranted.

So far, five phase II trials using chemotherapy, cetuximab and mostly IMRT are reported. Apart from Pfister et al., all of them were completed as scheduled and did not observe any unexpected toxicity [28-30,33], hence the combination of chemotherapy, cetuximab, and modern radiotherapy techniques seems feasible and the use of IMRT in a standard concomitant boost concept to reduce overall treatment time and reduce tumor cell repopulation [4,6,19] is practicable. Long-expected results of the randomized RTOG 0522 phase III trial comparing radiochemotheray +/- cetuximab have recently been presented: so far, there is no significant difference between the two treatment arms could be found with regard to progression-free survival and overall survival. There seems to be a trend toward higher distant-metastasis-free survival in the experimental arm (radiochemoimmunotherapy). Many patients have not yet even reached median follow-up, subgroup analyses regarding the influence of radiotherapy technique (accelerated radiotherapy (3D) vs IMRT) has not yet been published and is yet unclear [35]. Therefore it is too early to draw any conclusions from this trial.

Unfortunately patient accrual to the REACH trial is rather slow reflecting the fact that treatment of locally advanced SCCHN is still primarily surgical in Germany. However, functional outcome in of conservative treatment strategies as reflected in the REACH trial is excellent, apart from the patient with distant progressive disease and mediastinal mass, treatment related dysphagia has rapidly recovered post treatment. To date, there has been no case of swallowing dysfunction.

## Conclusion

The combination of our standard radiochemotherapy regimen with carboplatin/5-FU and cetuximab is feasible resulting in promising objective response rates. Also the use of a classical concomitant boost concept in intensity-modulated technique has been shown to be practicable in a routine clinical setting resulting in only moderate overall toxicity of the regimen. However, patient accrual needs to be improved.

## Competing interests

JD is a member of the Merck KGaA advisory board. All other authors declare that they have no competing interests.

Merck Serono has reviewed the publication, views and opinions described do not necessarily reflect those of Merck Serono.

## Authors' contributions

ADJ, JK, CS, and MWM are responsible for patient accrual, ADJ, AVN, KP, MWM for patient treatment, ADJ, JK, CS, and MWM for patient follow-up. MWM and JD were responsible for concept and design of the trial. ADJ, AVN, and KL coordinated and organized the trial. All authors read and approved the final manuscript.

## Financial support

Supported by a project grant from Merck KGaA, Darmstadt, Germany
